# Consensus clustering and development of a risk signature based on trajectory differential genes of cancer-associated fibroblast subpopulations in colorectal cancer

**DOI:** 10.1007/s00432-024-05906-z

**Published:** 2024-08-09

**Authors:** Ke Yu, Jiao Wang, Yueqing Wang, Jiayi He, Shangshang Hu, Shougang Kuai

**Affiliations:** 1 Wuxi Huishan District People’s Hospital, No. 2, Zhan Qian North Road, Luoshe Town, Huishan District, Wuxi City, Jiangsu Province China; 2 Affiliated Huishan Hospital of Xinglin College, Nantong University, Wuxi, Jiangsu China; 3 Southeast University, Nanjing, China

**Keywords:** Cancer-associated fibroblasts (CAFs), Colorectal cancer (CRC), Prognostic

## Abstract

**Background:**

Cancer-associated fibroblasts (CAFs) play a crucial role in the progression of colorectal cancer (CRC). However, the impact of CAF subpopulation trajectory differentiation on CRC remains unclear.

**Methods:**

In this study, we first explored the trajectory differences of CAFs subpopulations using bulk and integrated single-cell sequencing data, and then performed consensus clustering of CRC samples based on the trajectory differential genes of CAFs subpopulations. Subsequently, we analyzed the heterogeneity of CRC subtypes using bioinformatics. Finally, we constructed relevant prognostic signature using machine learning and validated them using spatial transcriptomic data.

**Results:**

Based on the differential genes of CAFs subpopulation trajectory differentiation, we identified two CRC subtypes (C1 and C2) in this study. Compared to C1, C2 exhibited worse prognosis, higher immune evasion microenvironment and high CAF characteristics. C1 was primarily associated with metabolism, while C2 was primarily associated with cell metastasis and immune regulation. By combining 101 combinations of 10 machine learning algorithms, we developed a High-CAF risk signatures (HCAFRS) based on the C2 characteristic gene. HCAFRS was an independent prognostic factor for CRC and, when combined with clinical parameters, significantly predicted the overall survival of CRC patients. HCAFRS was closely associated with epithelial-mesenchymal transition, angiogenesis, and hypoxia. Furthermore, the risk score of HCAFRS was mainly derived from CAFs and was validated in the spatial transcriptomic data.

**Conclusion:**

In conclusion, HCAFRS has the potential to serve as a promising prognostic indicator for CRC, improving the quality of life for CRC patients.

**Supplementary Information:**

The online version contains supplementary material available at 10.1007/s00432-024-05906-z.

## Introduction

Colorectal cancer (CRC) is a common malignant tumor with the third highest incidence and the second highest mortality rate (Sung et al. 2020). Studies have shown significant heterogeneity among CRC patients, with significant differences in treatment and prognosis even among patients with the same clinical stage (Khaliq et al. 2022). This is mainly because we have overlooked the link between clinical pathological features and molecular characteristics (Hashimoto et al. 2022). Therefore, there is a need to increase our understanding of the molecular heterogeneity of CRC and contribute to clinical decision-making and the development of rational treatment strategies.

Currently, several research groups have proposed different subtypes of CRC (Lin et al. 2021; Guinney et al. 2015; Song et al. 2021). However, these CRC subtypes are based on bulk RNA sequencing, which fundamentally lacks the exploration of differences in the tumor microenvironment (TME) at the cellular level. With the development of biotechnology, single-cell RNA-seq has become a powerful tool for exploring tumor heterogeneity (Shaw et al. 2021). Cancer-associated fibroblasts (CAFs) are the main components of the TME stroma and play a crucial role in CRC metastasis and immune therapy resistance (Huang et al. 2022). Numerous research results have indicated that CAFs may be key driving factors for the clinical outcome of CRC (Kobayashi et al. 2022). CAFs are composed of multiple dynamic subgroups, each with different biological characteristics and functions (Lavie et al. 2022). Therefore, it is necessary to explore the dynamic process of CAFs subgroups and their impact on CRC classification.

In this study, we attempted to perform consensus clustering of CRC based on differentially expressed genes related to the differentiation trajectory of CAFs subgroups. We further developed and validated a new risk signature using machine learning to predict the prognosis of CRC patients, aiming to improve the quality of life for CRC patients.

## Materials and methods

### Data acquisition and processing

The single-cell sequencing dataset (GSE200997) (Khaliq et al. 2022) for this study was obtained from the Gene Expression Omnibus (GEO) database, which included 16 tumor samples and 7 normal samples. The R software packages "Seurat," "harmony," "stringr," and "SCP" were used for quality control, batch correction, and visualization of the single-cell sequencing data (Stuart et al. 2019; Aran et al. 2019). The following filtering criteria were applied to the single-cell data: genes expressed in less than 3 single cells and cells expressing fewer than 100 genes were excluded, as well as cells expressing more than 5% mitochondrial genes. In terms of cell annotation, the SingleR package was utilized for preliminary annotation, followed by subsequent correction based on the CellMarker2.0 database (Hu et al. 2023). Differential gene expression analysis between normal fibroblasts and cancer-associated fibroblasts (CAFs) was performed using a threshold of log2FC ≥ 0.585 and adjusted p-value < 0.05. Monocle 2 package (Qiu et al. 2017) was used for pseudotime trajectory analysis of the single cells.

The bulk RNA sequencing data for this study was obtained from The Cancer Genome Atlas (TCGA) database, specifically the TCGA-COAD and TCGA-READ cohorts, which were integrated into a TCGA-CRC cohort. Additionally, three CRC cohorts from the GEO database (GSE17538 (Chen et al. 2019), GSE39582 (Marisa et al. 2013), GSE29621 (Chen et al. 2012)) were included. Further details of the datasets can be found in Supplementary Table 1. During the data processing phase of this study, the normalizeBetweenArrays function from the Limma package was utilized for data normalization. Batch effect removal was conducted using the ComBat function from the sva package.

Spatial transcriptomics (ST) data from Wu et al.'s study (Wu et al. 2022) were processed using the "Seurat" R package. Dimensionality reduction of the ST data was performed using the RunPCA function, followed by clustering of similar ST spots using the FindNeighbors and FindClusters functions. Preliminary annotation of the different clusters was based on hematoxylin and eosin (H&E) staining and unsupervised clustering analysis.

### Cell communication

Cell–cell crosstalk relationships were analyzed using the "CellChat" package in R software, and ligand-receptor interactions between them were predicted. Seurat-normalized data were used as input for Cellchat, choosing CellChatDB.human as the database for ligand-receptor interactions. Communication probabilities were determined using the computeCommunProb function, which disclosed both the number and significance of cellular interactions. Visualization of the communication dynamics among ligand-receptor (L-R) pairs was achieved through the netVisual_bubble function (Jin et al. 2021).

### Gene set scoring and deconvolution algorithm

Gene set scoring for single-cell and ST sequencing data was performed using the "AddModuleScore" function in the "Seurat" R package. The "ssGSEA" algorithm, part of the "GSVA" R package, was utilized to carry out deconvolution scoring of bulk sequencing data (Hänzelmann et al. 2013). This deconvolution approach has been extensively applied in a variety of studies previously published (Tan et al. 2023; Gui et al. 2024; Liu et al. 2022).

### Consensus unsupervised clustering analysis

The "ConsensusClusterPlus" (Wilkerson and Hayes 2010) package in R was used for consensus unsupervised clustering analysis. For each dataset sample, molecular subtyping was conducted using ConsensusClusterPlus version 1.52.0. The Pam method and Spearman distance metric were employed to execute 500 bootstraps, each comprising at least 80% of the dataset specimens. The range for the number of clusters (k) was set between 2 and 10, with the optimal k determined based on the cumulative distribution function (CDF) and the area under the curve (AUC).

### Prognostic analysis

The "survival" and "survminer" R packages were used for prognostic evaluation in this study, including univariate/multivariate Cox analysis and Kaplan–Meier survival curve plotting.

### Gene ontology (GO) and Kyoto encyclopedia of genes and genomes (KEGG) analysis

GO and KEGG analysis were performed using the "clusterProfiler" package and the "org.Hs.eg.db" package in R software. Adjusted p-value < 0.05 and adjusted q-value < 0.05 were considered statistically significant.

### Calculation of CAFs abundance and identification of CAFs markers

CAF abundance in the tumor microenvironment was evaluated using two algorithms, MCPcounter (Racle et al. 2017) and EPIC (Racle and Gfeller 2020), based on the "MCPcounter" and "EPIC" packages in R software. The third algorithm for calculating CAF abundance utilized the Tumor Immune Dysfunction and Exclusion (TIDE) online tool (http://tide.dfci.harvard.edu/) (Fu et al. 2020). CAF-related markers in this study (LUM, DCN, COL1A1, COL1A2, FAP, PDPN, PDGFRA, PDGFRB, S100A4, ACTA2, VIM, TGFB1) were obtained from a previous study (Han et al. 2020).

### Weighted Gene Co-expression network analysis (WGCNA)

The technique of Weighted Gene Co-expression Network Analysis (WGCNA) is extensively applied for the identification of gene expression modules within biological processes (Langfelder and Horvath 2008). Utilizing the WGCNA package, this research analyzed the co-expression networks of genes in conditions C1 and C2. A scale-free network was established by adopting a soft thresholding power of 3. Module detection was carried out through the dynamic tree cut method. For the accurate identification of gene modules significantly related to C1 and C2, those modules demonstrating the strongest correlations were chosen for in-depth analysis.

### Gene set variation analysis (GSVA)

The limma, GSEABase, GSVA, pheatmap, and ggplot2 packages in R software were used for GSVA and visualization. The "HALLMARK" and "KEGG" gene sets were obtained from the Molecular Signatures Database (https://www.gsea-msigdb.org/gsea/msigdb).

### Tumor microenvironment correlation analysis

The estimate (Yoshihara et al. 2013) and limma packages in R software were used to evaluate the differences in tumor microenvironment scores between C1 and C2, including estimate, immune, tumor purity, and stromal scores. The CIBERSORT package (Newman et al. 2015) in R software was used to assess the differences in immune cell infiltration between C1 and C2 in the tumor immune microenvironment. The infiltrating score of 16 immune cells and the activity of 13 immune-related pathways were determined using the ssGSEA function of the "gsva" R package.

### Immune escape and mutation analysis

The TIDE tool was used to evaluate the immune escape scores between C1 and C2. To identify gene mutation features between C1 and C2, waterfall plots were generated using the "maftools" package.

### Development and validation of risk signatures based on machine learning

In this investigation, the leave-one-out cross-validation (LOOCV) methodology, as formulated by Zaoqu Liu et al. (2022), was harnessed for the derivation of risk signatures. This methodology amalgamated ten distinct machine learning algorithms, namely generalized boosted regression modeling (GBM), Lasso, Ridge, random survival forests (RSF), supervised principal component analysis (SuperPC), stepwise Cox regression, CoxBoost, partial least squares Cox regression (plsRcox), elastic net (Enet), and survival support vector machine (survival SVM), culminating in an aggregate of 101 possible algorithmic permutations. The optimal permutation was determined through the evaluation of the concordance index (C-index) across these 101 combinations. This study fitted the training set (TCGA-CRC) and validated it using three independent datasets (GSE17538, GSE39582, GSE29621). The optimal algorithm combination was determined based on the average C-index of the four datasets.

### Construction of nomogram model

The nomogram model was constructed based on multifactor Cox regression analysis using the "rms" package. Calibration curves were used to predict the survival rate of CRC patients. Time-dependent ROC curves and decision curve analysis (DCA) were used to evaluate the performance of variables in the nomogram. Time-dependent ROC curves and DCA were plotted using the "timeROC" and "ggDCA" packages, respectively.

### Collection and comparison of risk signature related to cancer-associated fibroblasts (CAFs)

As of May 2024, this study conducted a systematic search for risk features related to CAFs using the keywords "(CAF OR CAFs OR Fibroblasts) colorectal cancer signature" in the PubMed database. A total of 9 articles were retrieved (Zheng et al. 2021, 2023a; Liang et al. 2023; Xu and Pan 2021; Wang et al. 2023; Lv et al. 2023; Zhang et al. 2022; Wei et al. 2024; Cai et al. 2024). The risk signature genes from these studies were collected. By matching the expression profiles and survival information of 5 colorectal cancer datasets (TCGA, GSE17538, GSE29621, GSE39582, Meta-cohort), the C-index of these risk signatures was calculated and compared with the signature of this study.

### Statistical analysis

All statistical analyses in this study were performed using R software version 4.2.0. Wilcoxon t-test was used to compare variables between groups. Spearman correlation analysis was used to evaluate the correlation between continuous variables. A P-value less than 0.05 was considered statistically significant (*P < 0.05, **P < 0.01, ***P < 0.001).

## Results

### High CAF is an adverse prognostic factor for CRC

**Fig. 1:**
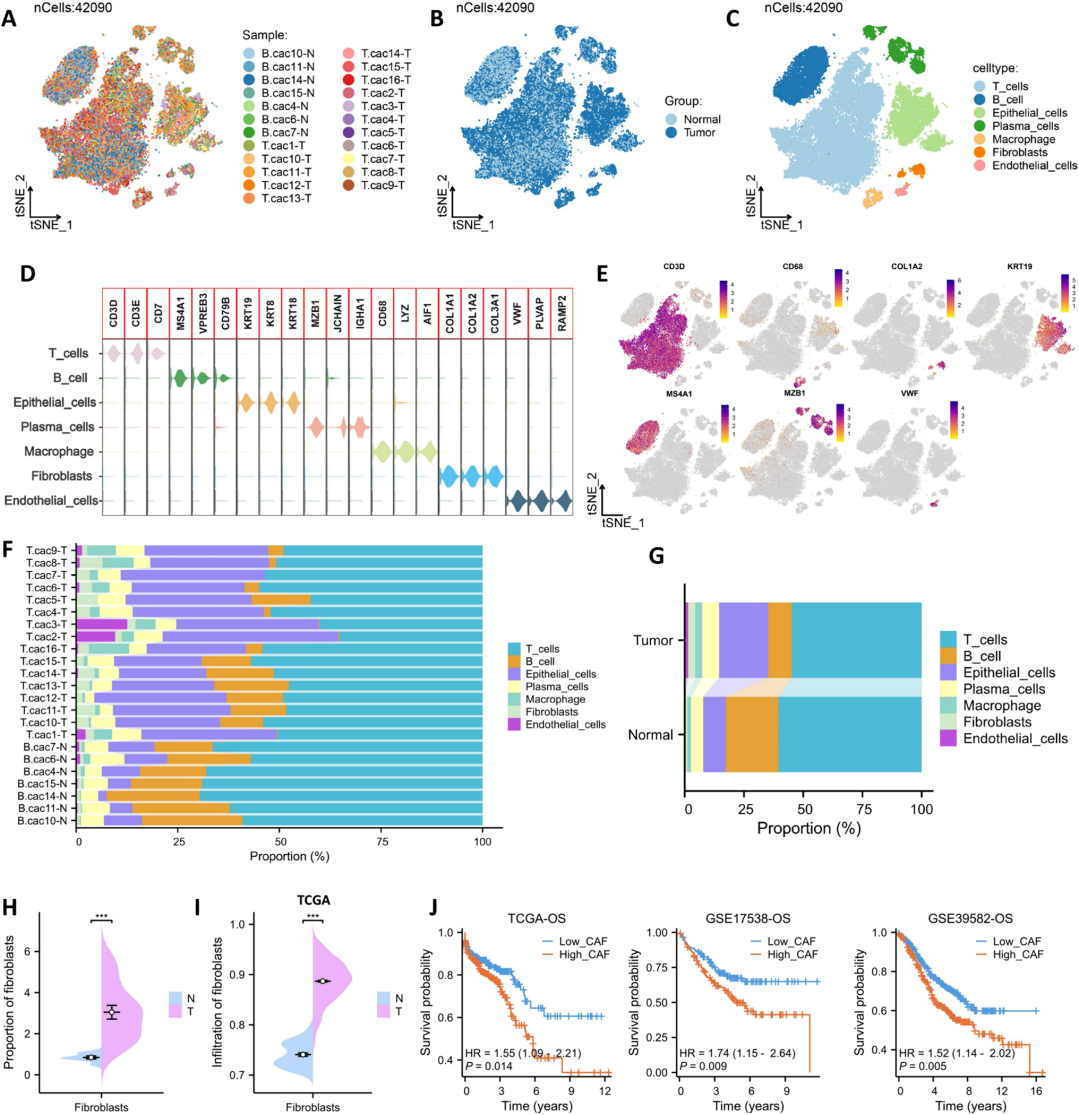
High CAF is an adverse prognostic factor for CRC. **A**. tSNE plots for each sample. **B**. tSNE plots for each group. **C**. tSNE plots for the seven cell types. **D**. Violin plots showing the expression of specific markers for each cell type. **E**. tSNE plots for the expression of specific markers for each cell type. **F**. Proportional distribution of the seven cell types in each sample. **G**. Proportional distribution of the seven cell types in the normal and tumor groups. **H**. Difference in the proportions of fibroblasts between the normal and tumor groups. **I**. Difference in fibroblast infiltration between the normal and tumor groups based on TCGA cohort. **J**. Difference in survival between the high CAF group and the low CAF group based on three CRC cohorts

The GSE200997 dataset comprises single-cell sequencing data for 7 normal colon tissues and 16 CRC tissues. Initially, the dataset included 49,859 cells; following quality control measures, 42,090 high-quality cells were obtained. The single-cell atlas for each sample and each group is shown in Fig. 1A, B. We identified 7 major cell types (Fig. 1C) with specific marker expression for each cell type (Fig. 1D, E). The proportions of each cell type varied among different samples, reflecting the heterogeneity among CRC patients (Fig. 1F). In the tumor group, the proportions of 5 cell types (epithelial cells, plasma cells, macrophages, fibroblasts, and endothelial cells) were higher than those in the normal group (Fig. 1G). Studies have shown that fibroblasts play a crucial role in tumor progression (Chhabra and Weeraratna 2023). In this study, the proportion of fibroblasts in the tumor group was significantly higher than that in the normal group (Fig. 1H). The infiltration level of fibroblasts in the bulk queue was estimated based on fibroblast markers, and similarly, the fibroblast infiltration in the tumor group was significantly higher than that in the normal group (Fig. 1I). Furthermore, based on three independent CRC cohorts, we found that the overall survival (OS) of CRC patients in the high CAF group was significantly lower than that in the low CAF group (Fig. 1J).

### Characteristics of cancer-associated fibroblasts (CAFs) in the tumor group

**Fig. 2:**
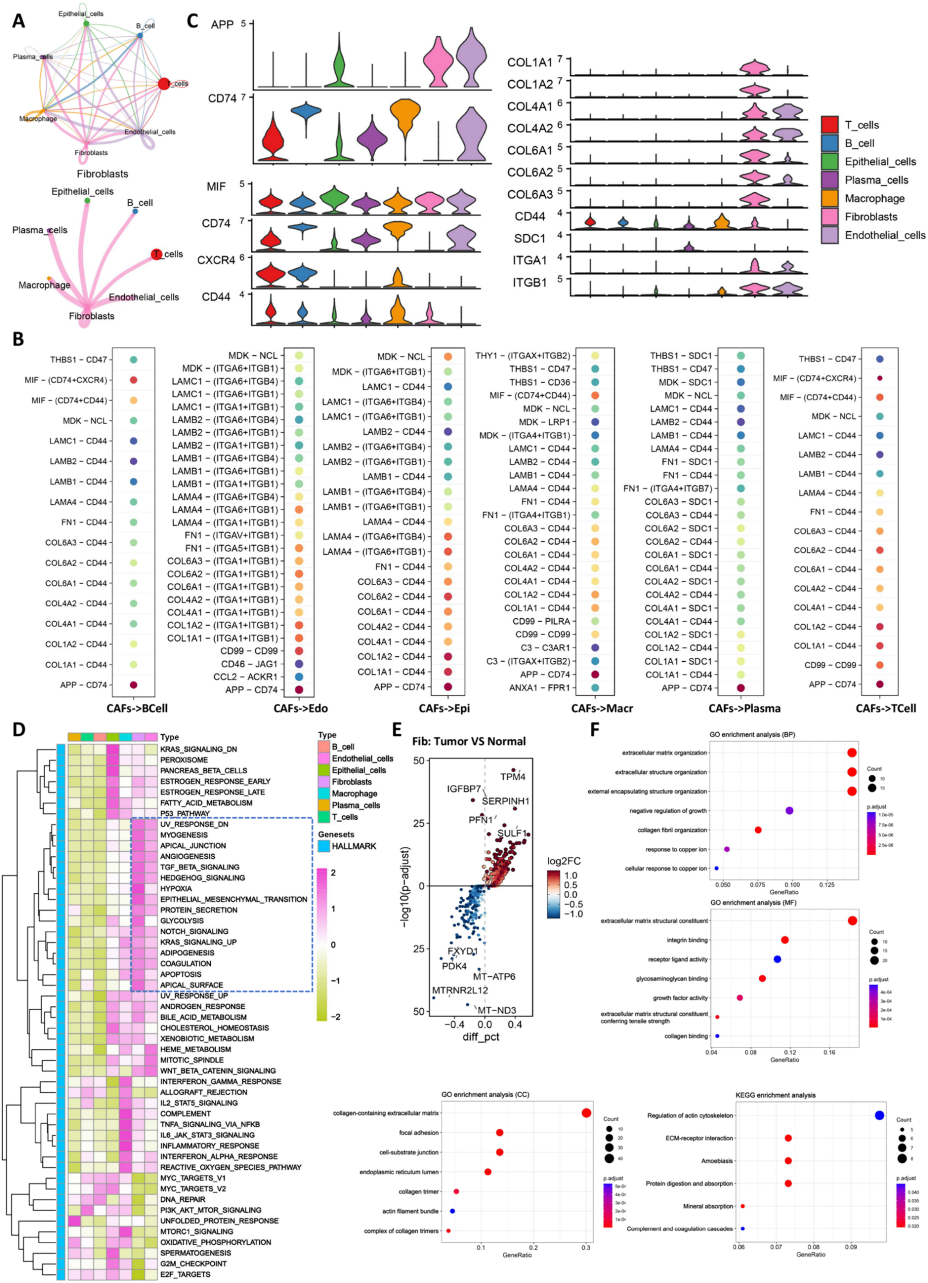
Characteristics of cancer-associated fibroblasts in the tumor group. **A**. Number of communications between CAFs and six cell types. **B**. Ligand-receptor interactions between CAFs and six cell types. **C**. Expression of APP pathway, MIF pathway, and collagen pathway in seven cell types. **D**. GSVA heatmap of the seven cell types. **E**. Volcano plot of differentially expressed genes between normal and tumor fibroblasts. **F**. GO/KEGG analysis

We analyzed the characteristics of CAFs in the tumor group. In terms of cell communication, we found that CAFs interact closely with the other six cell types (Fig. 2A). There are multiple ligand-receptor pairs that exhibit strong communication between them (Fig. 2B). For example, the ligands of the APP pathway, MIF pathway, and collagen pathway are highly expressed in CAFs (Fig. 2C). GSVA analysis showed that CAFs exhibit distinct features such as epithelial-mesenchymal transition, hypoxia, glycolysis, and angiogenesis compared to other cell types (Fig. 2D). Furthermore, we obtained differentially expressed genes between normal and tumor fibroblasts and performed GO/KEGG analysis (Fig. 2E), revealing that these genes are mainly enriched in terms related to extracellular matrix remodeling (Fig. 2F).

### Heterogeneity and trajectory differentiation of CAF subgroups in the tumor group

**Fig. 3:**
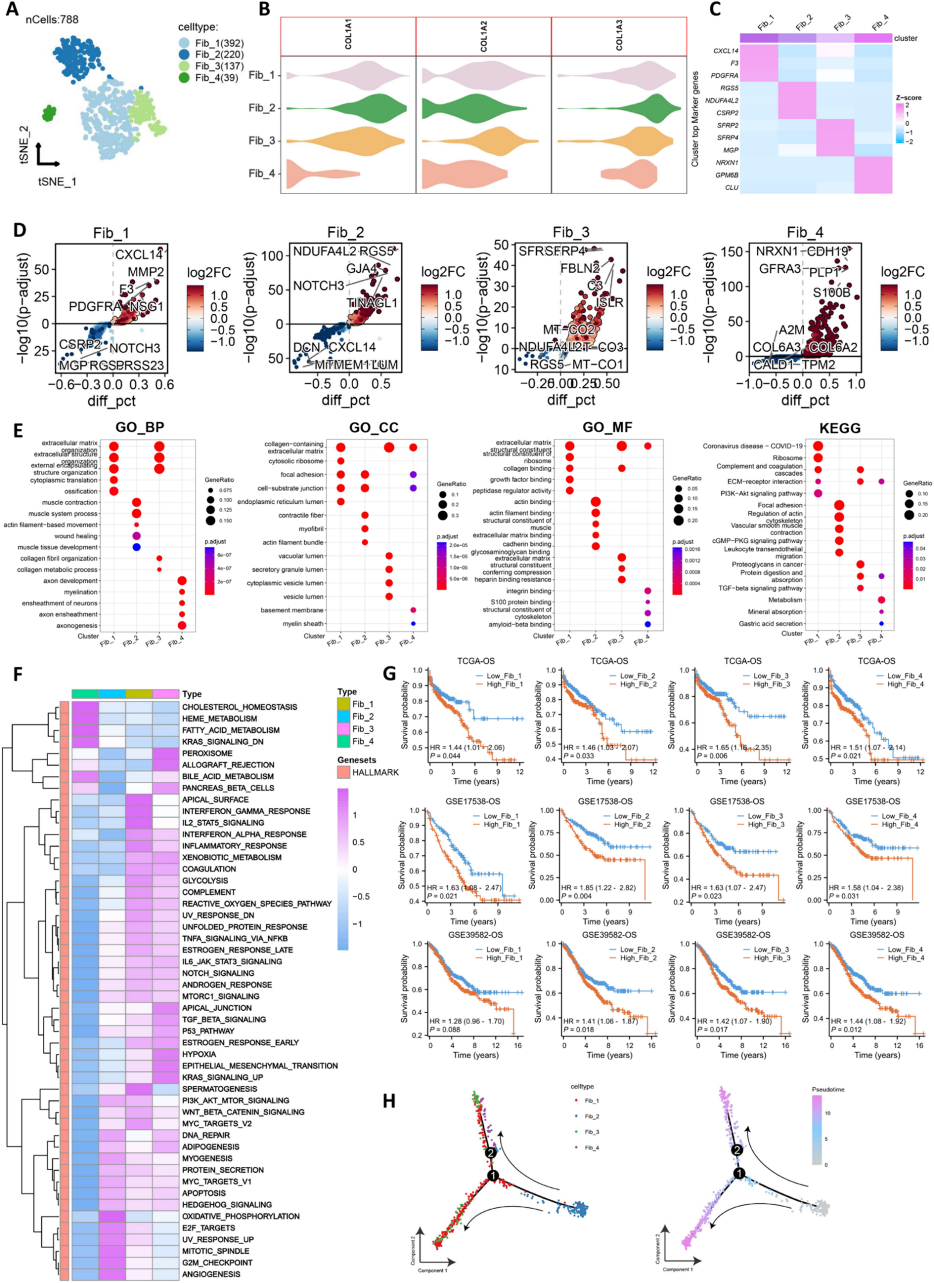
Heterogeneity and trajectory differentiation of CAF subgroups in the tumor group. **A**. tSNE plot of CAF subgroups. **B**. Expression levels of three CAF markers in CAF subgroups. **C**. Heatmap showing the expression of specific markers in the four CAF subgroups. **D**. Volcano plot of differentially expressed genes in the four CAF subgroups. **E**. GO/KEGG analysis. **F**. GSVA heatmap of the four CAF subgroups. **G**. Overall survival curves of the four CAF subgroups. **H**. Pseudotime analysis

We further subgrouped the fibroblasts in the tumor group and identified four distinct CAF subgroups (Fib_1-Fib_4) (Fig. 3A). All four subgroups express markers of CAFs (COL1A1, COL1A2, and COL1A3) (Fig. 3B). Fib_1 specifically expresses CXCL14, F3, and PDGFRA; Fib_2 specifically expresses RGS5, NDUFA4L2, and CSRP2; Fib_3 specifically expresses SFRP2, SFRP4, and MGP; Fib_4 specifically expresses NRXN1, GPM6B, and CLU (Fig. 3C). We obtained differentially expressed genes between these four cell types and performed GO/KEGG analysis (Fig. 3D), revealing significant heterogeneity among them (Fig. 3E). GSVA analysis also supported this finding (Fig. 3F). Survival analysis showed that all four CAF subgroups are adverse prognostic factors in CRC (Fig. 3G). Since CAF subgroups undergo dynamic differentiation, we further conducted pseudotime analysis. We found that Fib_2 represents the starting population of differentiation, Fib_4 represents the intermediate population, while Fib_1 and Fib_3 represent the terminal differentiated populations (Fig. 3H). In summary, CAF subgroups exhibit significant heterogeneity, and each subgroup is associated with poor prognosis in CRC. Therefore, the dynamic differentiation process of CAF subgroups may play an important role in the clinical outcomes of CRC.

### Development and validation of CRC subtypes

**Fig. 4:**
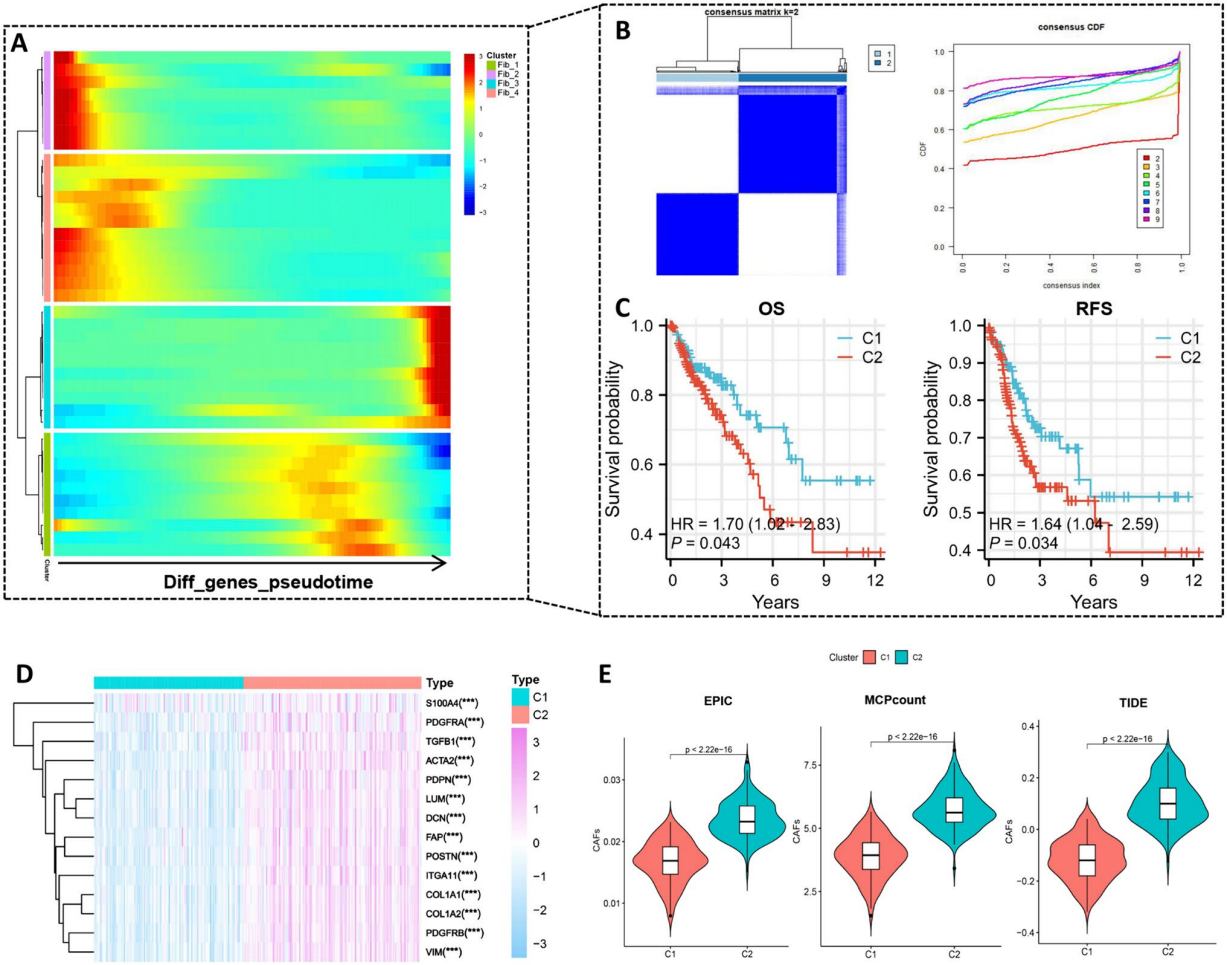
Development of CRC subtypes. **A**. Heatmap of pseudotime analysis of CAF subgroups. **B**. Classification of TCGA CRC cohort into two subtypes when k = 2. **C**. Overall survival (OS) and recurrence-free survival (RFS) analysis between C1 and C2. **D**. Heatmap showing differential expression analysis of CAF markers between C1 and C2. **E**. Differential analysis of CAF abundance between C1 and C2 based on three CAF abundance algorithms

Based on pseudotime analysis of CAF subgroups, we identified 1929 differentially expressed genes during CAF subgroup differentiation (Fig. 4A), of which 282 genes were found to have prognostic significance (Supplementary Table 2). Using the expression profiles of these 282 genes, we performed consensus clustering analysis to classify CRC samples. All samples were divided into k (2–9) clusters, but k = 2 was determined to be the optimal number, defining the clusters as C1 and C2 (Fig. 4B). Prognostic analysis of overall survival (OS) and recurrence-free survival (RFS) showed that C2 had worse prognosis compared to C1 (Fig. 4C). Furthermore, we obtained 14 CAF markers from previous studies and found that all markers were expressed at higher levels in C2 (Fig. 4D). We also calculated the CAF abundance in each sample of the TCGA cohort using three algorithms (EPIC, MCPcount, TIDE) and found that C2 had significantly higher CAF abundance compared to C1 (Fig. 4E). Therefore, C1 was characterized by low CAF features, while C2 was characterized by high CAF features. Additionally, we validated the reliability and stability of C1 and C2 subtypes using two external independent CRC datasets (GSE17538 and GSE39582) (Supplementary Fig. 1A–D).

### C1 and C2 exhibit significant heterogeneity

**Fig. 5:**
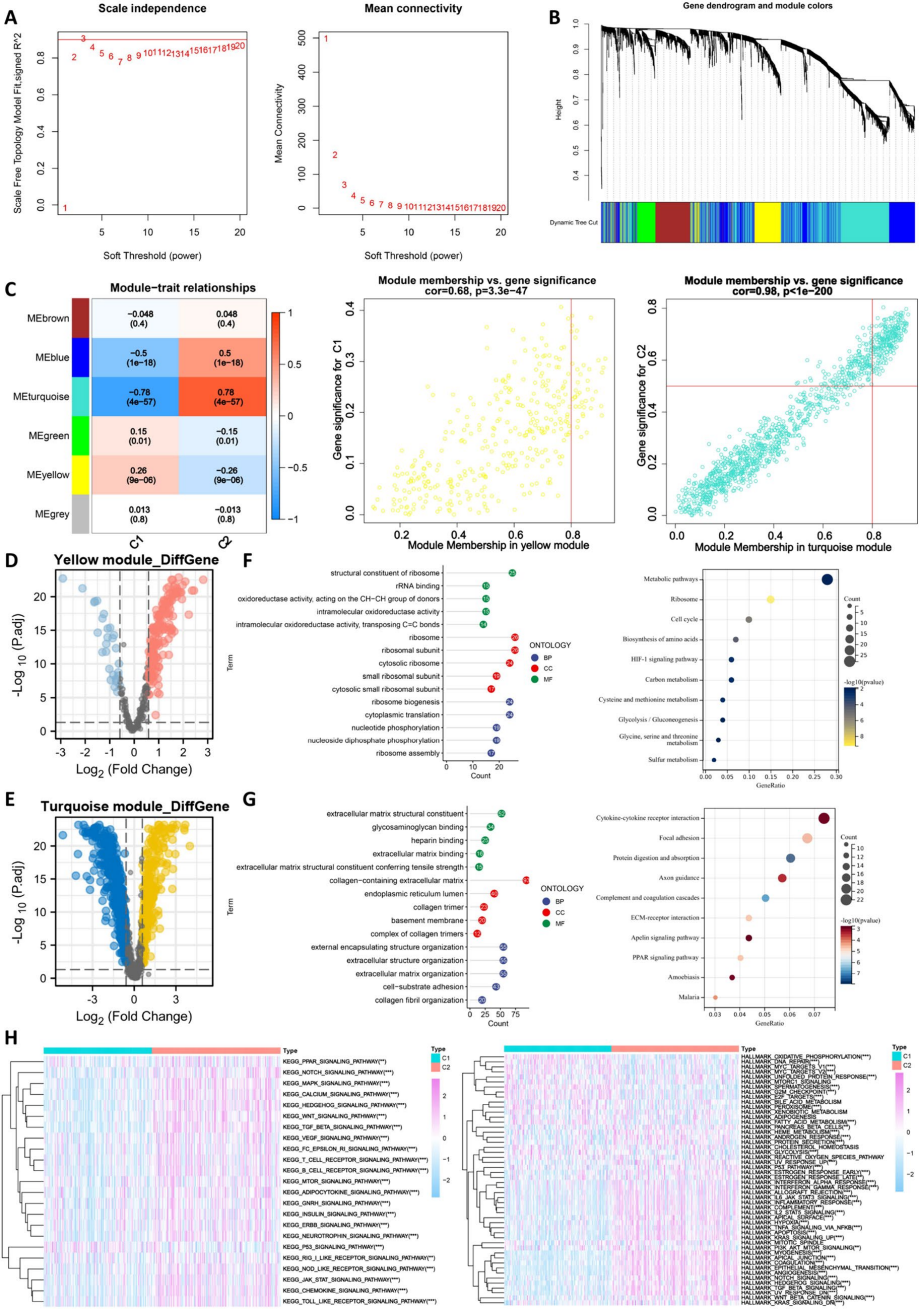
Exploring the heterogeneity of biological characteristics between C1 and C2. **A**. Soft threshold used for WGCNA calculation. **B**. Dendrogram of the six modules. **C**. Heatmap and scatter plot showing the correlation of module genes with CRC subtypes. **D**. Volcano plot showing differential gene analysis in the yellow module between normal and tumor groups. **E**. Volcano plot showing differential gene analysis in the turquoise module between normal and tumor groups. **F**. GO and KEGG analysis of differentially expressed genes in the yellow module. **G**. GO and KEGG analysis of differentially expressed genes in the turquoise module. **H**. GSVA showing the top 15 differentially regulated pathways in CRC subtypes

This study further analyzed the heterogeneity between C1 and C2. The results from the ESTIMATE algorithm showed that C2 had higher StromalScore, ImmuneScore, and ESTIMATEscore, and lower tumor purity score compared to C1 (Supplementary Fig. 2A). Therefore, C2 may have more immune cell infiltration. The immune cell infiltration results showed that the levels of CD8 + T cells and plasma cells were significantly higher in C1 compared to C2, while the levels of macrophages (M0 and M2) in C2 were significantly higher than those in C1(Supplementary Fig. 2B). We further used the ssGSEA algorithm to measure the infiltration scores of 16 immune cells and the activity of 13 immune-related pathways. The results showed that CD8 + T cells were significantly higher in C1 compared to C2, while immune suppressor cells, immune checkpoint pathways, and inflammation-related pathways were significantly higher in C2 compared to C1 (Supplementary Fig. 2C). Therefore, compared to C1, C2 is in an immune-suppressed state and may have stronger immune escape ability. We evaluated the immune escape ability of C1 and C2 using the tumor immune dysfunction and exclusion (TIDE) algorithm, and the results showed that C2 had stronger T cell exclusion, T cell dysfunction, and TIDE scores (Supplementary Fig. 2D). These results were validated in two independent CRC cohorts (GSE17538 and GSE39582) (Supplementary Fig. 3A, B). We further evaluated the mutation differences between C1 and C2. As shown in Supplementary Fig. 2E, there were differences in APC, TTN, RYR2, and DNAH5 among the top 19 mutated genes between C1 and C2. Using the WGCNA approach based on the TCGA cohort, we identified the characteristic genes of C1 and C2. The optimal soft threshold was β = 3, with an R2 value of 0.9, allowing the formation of a scale-free network (Fig. 5A). We then performed cluster analysis on the modules and identified six modules (Fig. 5B), among which the yellow and turquoise modules showed the strongest correlation with C1 and C2, respectively (Fig. 5C). Next, we performed differential analysis on the genes in the yellow and turquoise modules using the normal and tumor groups from the TCGA cohort. The yellow module had 189 differentially expressed genes (Fig. 5D), which we defined as the characteristic genes of C1, while the turquoise module had 623 differentially expressed genes, which we defined as the characteristic genes of C2 (Supplementary Table 3) (Fig. 5E). GO and KEGG analysis showed that C1 was mainly associated with metabolic regulation (Fig. 5F), while C2 was mainly associated with extracellular matrix, chemokines, and cell adhesion (Fig. 5G). In addition, GSVA results showed that the upregulated pathways in C1 were related to metabolism and cell cycle regulation, while the upregulated pathways in C2 were related to metastasis, angiogenesis, and cell immune regulation (Fig. 5H).

### Development and validation of High-CAF risk signatures (HCAFRS)

**Fig. 6:**
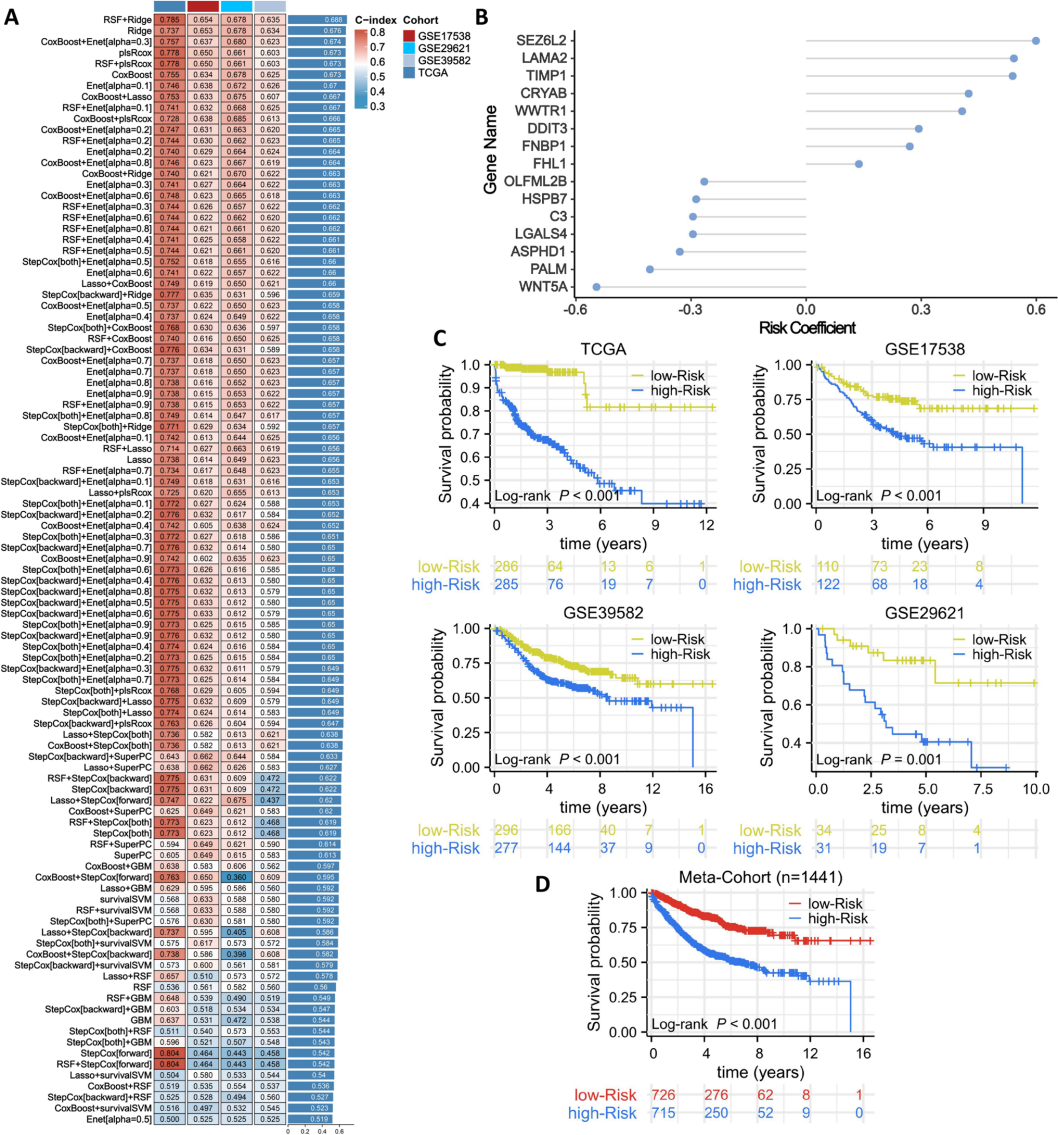
Development and validation of risk signatures. **A**. Fitting of the training set (TCGA) using 101 combinations of 10 machine learning algorithms, followed by validation on three independent datasets (GSE17538, GSE39582, GSE29621) and calculation of the average C-index for these four datasets. **B**. 20 risk genes and their corresponding risk coefficients. **C**. Overall survival curves for the training set (TCGA) and validation sets (GSE17538, GSE39582, GSE29621). **D**. Overall survival curve for the Meta-Cohort (combined training and validation sets)

Based on the expression profiles of 623 C2 characteristic genes in the TCGA cohort, a univariate Cox analysis identified 118 prognostic genes (Supplementary Table 4). These 118 prognostic genes were fitted to the training set (TCGA) using 101 combinations of 10 machine learning algorithms, followed by validation on three independent datasets (GSE17538, GSE39582, GSE29621) and calculation of the average C-index for these four datasets. We found that the RSF + Ridge algorithm combination had the highest average C-index (0.688) (Fig. 6A). The RSF + Ridge algorithm combination identified 15 risk genes and their corresponding risk coefficients (Fig. 6B). We weighted the expression of these 15 risk genes by their respective risk coefficients to obtain a risk score for each sample. Given that this risk signature was developed based on the C2 characteristic genes, and C2 exhibits high CAF features, we define it as the High-CAF Risk Signature (HCAFRS). Using the median value of HCAFRS, we divided the samples into high-risk and low-risk groups. The OS curves showed that the high-risk group had significantly lower survival times than the low-risk group in the training set (TCGA) and validation sets (GSE17538, GSE39582, GSE29621) (Fig. 6C). We combined the training and validation sets into a Meta-Cohort (n = 1441), which also showed the same trend (Fig. 6D).

### Construction of a columnar diagram model based on HCAFRS and clinical parameters as a powerful tool for predicting CRC prognosis

**Fig. 7:**
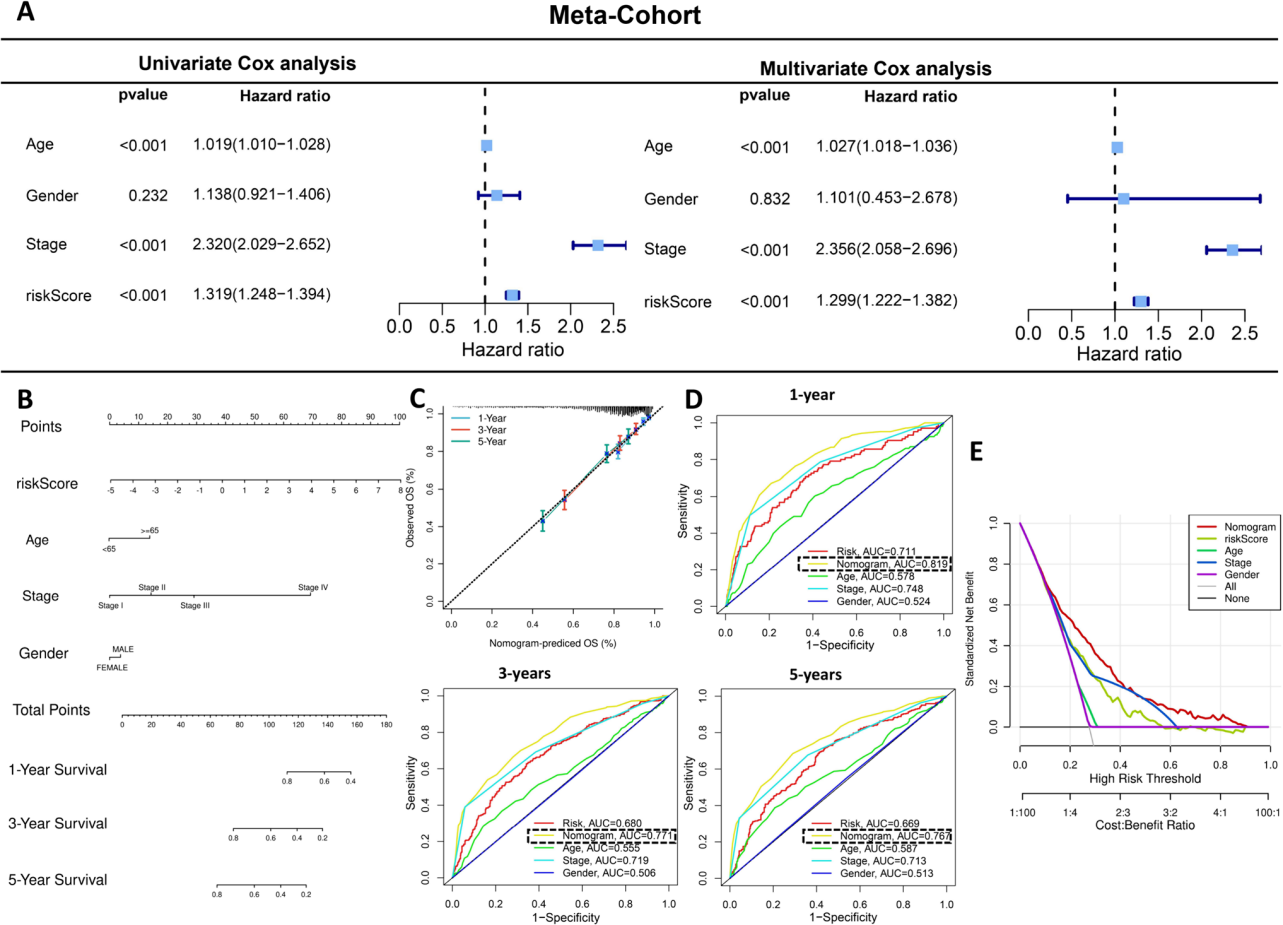
The nomogram model based on HCAFRS and clinical parameters as a powerful tool for predicting CRC prognosis. **A**. Univariate/multivariate Cox analysis of the Meta-Cohort. **B**. Nomogram model based on HCAFRS and clinical parameters. **C**. Calibration curves for 1, 3, and 5 years. **D**. Time-dependent ROC curves for 1, 3, and 5 years. **E**. Decision curve analysis (DCA)

We first performed univariate and multivariate Cox analyses on the training set and three validation sets, and found that in all four cohorts, HCAFRS risk score was an independent prognostic factor for CRC (Supplementary Fig. 4A–D). The Meta-Cohort also showed the same result (Fig. 7A), indicating the robustness and reliability of HCAFRS in predicting CRC prognosis. Stage, age, and gender are commonly used clinical parameters for CRC, therefore, we constructed a nomogram model based on these three clinical parameters and HCAFRS (Fig. 7B). The calibration curves demonstrated the high reliability of this nomogram model in predicting the overall survival of CRC at 1, 3, and 5 years (Fig. 7C). The time-dependent ROC curves showed that the columnar diagram model outperformed the other three clinical parameters at 1, 3, and 5 years (Fig. 7D). DCA further confirmed the reliability of this nomogram model (Fig. 7E). Therefore, the nomogram model based on HCAFRS and clinical parameters is a powerful tool for predicting CRC prognosis.

### Investigation of the biological characteristics of HCAFRS

**Fig. 8:**
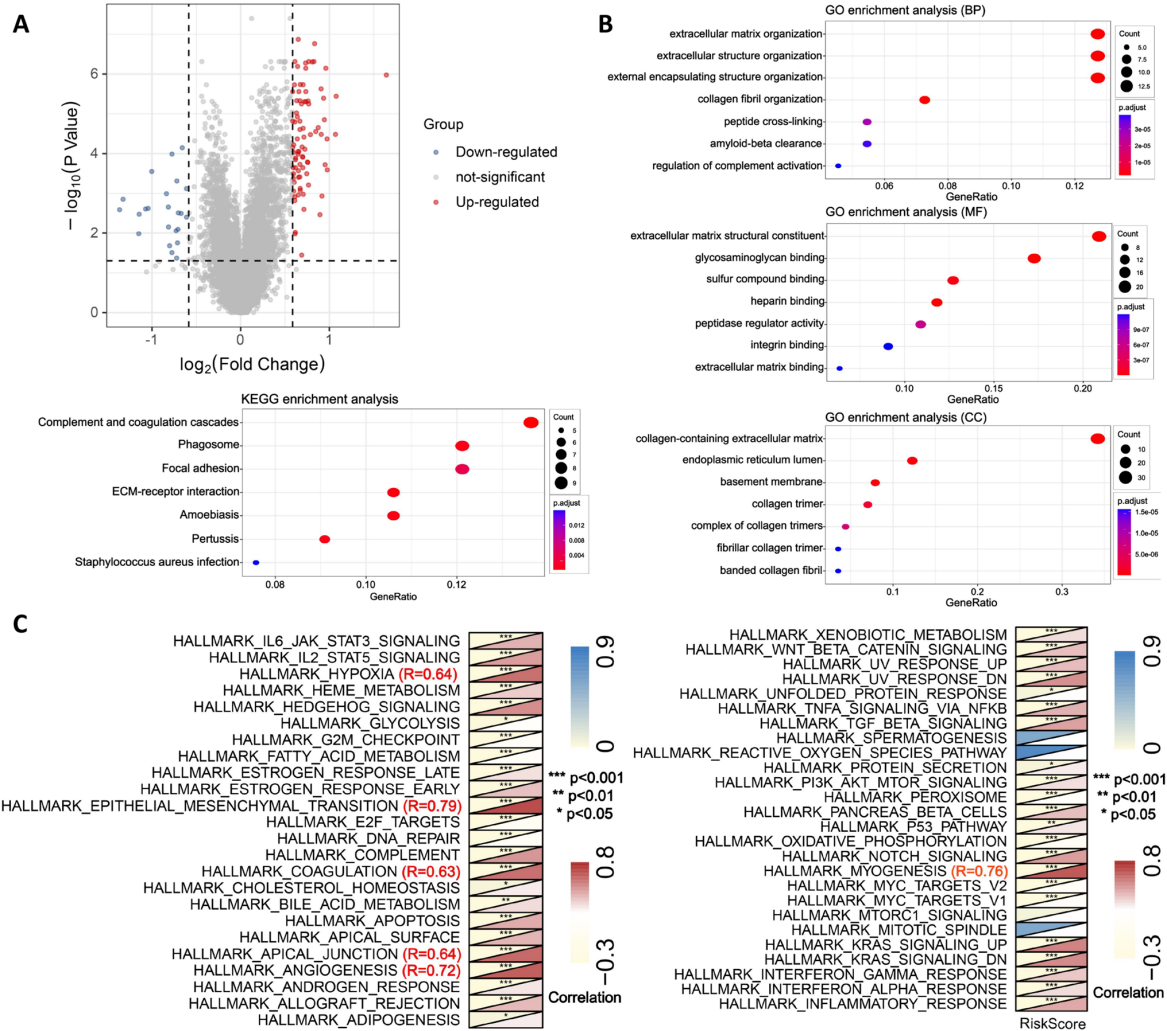
Biological characteristics of HCAFRS. **A**. Volcano plot showing differential gene expression between the high-risk and low-risk groups of HCAFRS. **B**. Heatmap showing the correlation of HCAFRS with GSVA. **C**. Scatter plot showing the correlation of HCAFRS risk score with hypoxia, epithelial-mesenchymal transition, apical junction, angiogenesis, and myogenesis

Based on the TCGA cohort, we identified 115 differentially expressed genes (logFC > 0.585, P < 0.05) between the high-risk and low-risk groups of HCAFRS (Fig. 8A). GO/KEGG analysis revealed that HCAFRS is associated with extracellular matrix remodeling (Fig. 8B). To explore the closely related biological features of HCAFRS, we performed gene set variation analysis (GSVA) on the TCGA cohort using the "hallmarker" gene set. We found that the risk score of HCAFRS was significantly positively correlated with hypoxia, epithelial-mesenchymal transition, apical junction, angiogenesis, and myogenesis (R > 0.6, P < 0.05) (Fig. 8C).

### CAF-mediated risk of HCAFRS

**Fig. 9:**
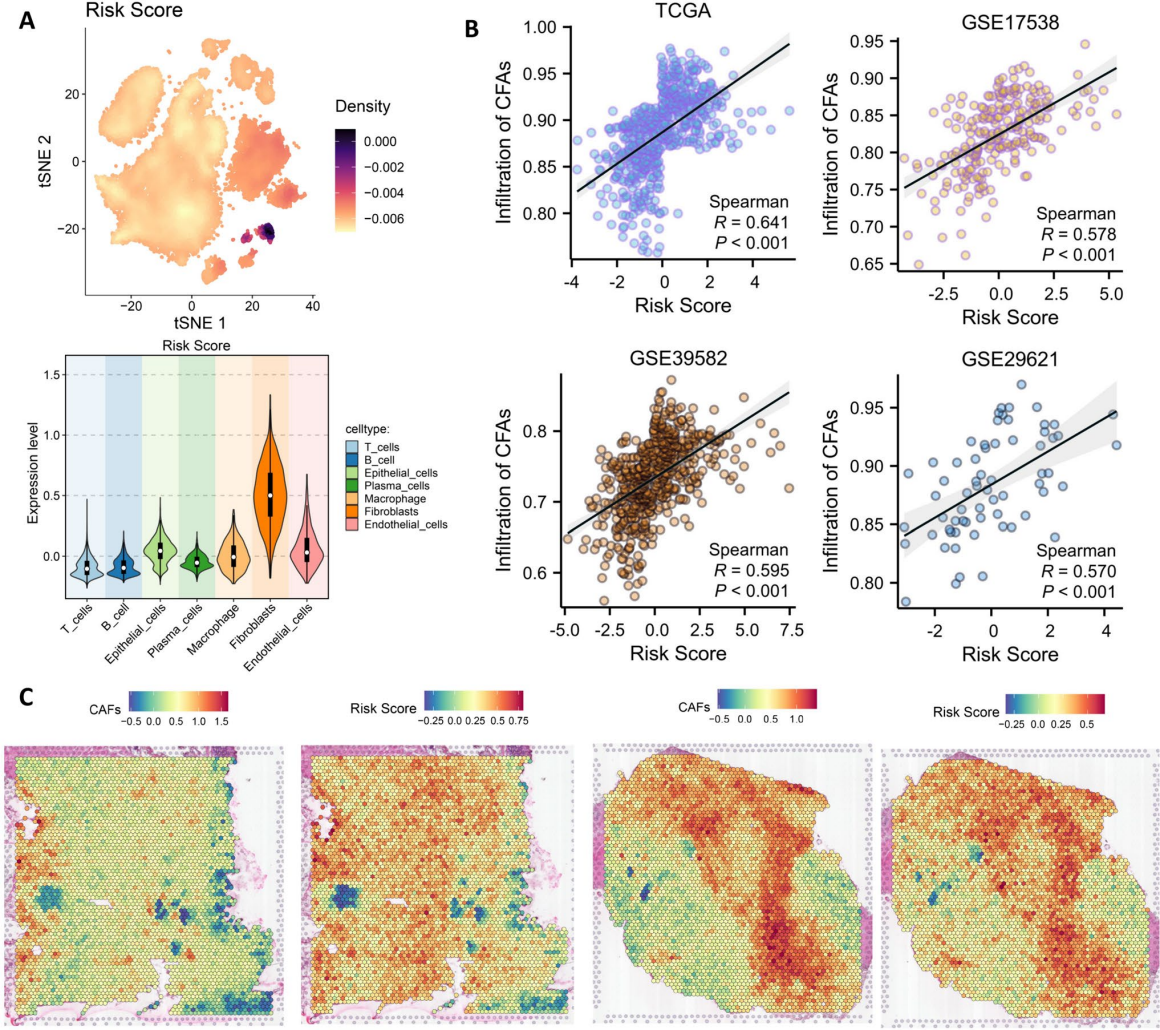
CAF-mediated risk of HCAFRS. **A**. tsne plot and differential analysis of the risk score of HCAFRS. **B**. Scatter plot showing the correlation between the risk score of HCAFRS and CAF infiltration based on four bulk sequencing datasets. **C**. Spatial feature plot of CAFs and the risk score of HCAFRS

Finally, we explored the relationship between HCAFRS and cancer-associated fibroblasts (CAFs). Single-cell data showed that HCAFRS is primarily expressed in the fibroblast cluster and exhibits significant differences compared to other cell clusters (Fig. 9A). Combining four bulk sequencing datasets, we found a significant positive correlation between the risk score of HCAFRS and the degree of CAF infiltration (R > 0.5, P < 0.05) (Fig. 9B). Furthermore, using two spatial transcriptomic datasets for validation, we observed a spatial overlap between the risk score of HCAFRS and the localization of CAFs (Fig. 9C). Therefore, the risk of HCAFRS is mainly derived from CAFs.

### The HCAFRS outperforms the previously published CAFs-related risk signature

A systematic search of existing studies revealed no similar approaches to constructing a colorectal cancer risk signature as the one presented in this study. The search was conducted using the keywords "CAF OR CAFs OR Fibroblasts colorectal cancer signature," resulting in the identification of 9 studies that built risk signatures based on CAFs. A comparison of this study with these 9 risk signatures (Supplementary Table 5) showed that the existing studies were primarily focused on constructing risk signatures using CAF markers or related genes and employed a single machine learning algorithm. Risk signatures based on CAF markers or related genes were found to be overly limited and overlooked the impact of subpopulation differentiation on CRC prognosis. Models fitted using a single machine learning algorithm demonstrated poor generalizability. Furthermore, in terms of CRC dataset utilization, this study utilized a more comprehensive CRC dataset for validation. The key aspect of a risk prognosis model lies in the stability of its prognostic performance. Initially, the risk genes from these 9 studies were collected (Supplementary Table 6), and subsequently, the Concordance Index (C-index) of this study's signature and the 9 signatures were calculated using 5 CRC datasets (TCGA, GSE17538, GSE29621, GSE39582, Meta-cohort) to compare their predictive capabilities for CRC survival prognosis. The risk gene expression profiles of Wang et al. and Lv et al. were not matched in the GSE39582 and Meta-cohort datasets. Therefore, in the GSE39582 and Meta-cohort datasets, the C-index of this study was compared with the remaining 7 signatures. The C-index results indicated that, except for the GSE29621 dataset, the signature of this study exhibited excellent predictive performance in the other 4 datasets (Supplementary Fig. 5). In conclusion, the approach of this study is more comprehensive, and the signature constructed based on consensus clustering of CAFs trajectory differential genes (HCAFRS) is superior to signatures constructed based on CAF markers or related genes.

## Discussion

The clear characteristics of CRC include strong heterogeneity, high metastatic rate, poor prognosis, and high recurrence rate (Sung et al. 2020). Previous studies have shown a close correlation between TME and CRC heterogeneity (Jackstadt et al. 2019), and CAFs in the TME are key drivers of CRC progression (Kobayashi et al. 2022). Studies have demonstrated that CAFs can promote drug resistance, metastasis, and immune escape in CRC cells (Gong et al. 2020). Currently, research has indicated that the heterogeneity of CAFs plays an important role in regulating the TME in CRC (Davidson et al. 2021). Recent studies have shown that single-cell transcriptomics partially reveals the heterogeneity of CRC influenced by CAFs. For example, in Qi et al.'s study, FAP^+^CAFs were identified as driving factors for immune escape and poor prognosis in CRC (Qi et al. 2022). In another study, INHBA^+^CAFs were associated with the prognosis and progression of CRC (Zheng et al. 2023b).

In this study, we clustered and categorized CAFs in the tumor group, and obtained four heterogeneous CAF subgroups (Fib_1-Fib_4). All four subgroups were identified as adverse prognostic factors for CRC patients. Previous studies have shown that CAFs in the TME are dynamic rather than static as the tumor progresses (Lavie et al. 2022). This study's proposed temporal analysis revealed that Fib_2 represents the differentiation initiating cell group, Fib_4 represents the intermediate differentiation cell group, while Fib_1 and Fib_3 represent the terminal differentiation cell group. Therefore, we speculate that the entire trajectory of CAFs may be closely related to the progression of CRC. In addition, quantified genes associated with CAFs are one of the factors promoting CRC progression (Kasashima et al. 2021). Therefore, analyzing the differentially expressed genes in the trajectory of CAFs is of great significance and provides a good foundation for distinguishing CRC subtypes.

We used consensus unsupervised clustering analysis to identify CRC subtypes based on differentially expressed genes in CAFs trajectories that have prognostic effects. We identified two CRC subtypes (C1 and C2). C2 had a worse prognosis compared to C1. C2 showed higher expression of CAF markers and higher CAF enrichment. Therefore, C1 exhibited low CAF characteristics while C2 exhibited high CAF characteristics. In terms of TME, compared to C1, C2 had lower tumor purity and higher immune and stromal scores, indicating more immune cell infiltration in C2. In terms of immune cell infiltration, compared to C1, C2 had higher infiltration of M2 macrophages. It is well known that M2 TAMs enhance CRC cell metastasis and inhibit T cells (Zhu et al. 2022). In this study, our results indicated that C2 had higher M2 TAM polarization, immune escape checkpoint scores, and significant upregulation of immune suppressive cells, providing a favorable environment for CRC metastasis and immune escape. We used the TIDE algorithm to simulate the two main mechanisms of immune escape, T cell exclusion, and T cell dysfunction (Fu et al. 2020). Based on the TIDE algorithm, we revealed that C2 had a higher potential for immune escape compared to C1. These results were independently validated in CRC cohorts.

Machine learning plays a significant role in biomedical research and clinical practice. By leveraging large-scale biomedical data and efficient algorithms, machine learning can provide accurate predictions to assist physicians in making better decisions and treatment plans (Swanson et al. 2023). Currently, there are many studies on constructing CRC risk signatures using machine learning, but most of them are based on a single machine algorithm and a single biological pathway. For example, risk signatures related to hypoxia constructed using LASSO regression analysis (Luan et al. 2022), risk signatures related to iron death (Shao et al. 2021), and risk signatures related to cell necrosis (Li et al. 2022). These features overlook other key biological information during CRC development, and a single machine algorithm is insufficient to better fit the genes in these risk signatures. In this study, we used WGCNA and differential analysis to obtain the feature genes of C2. The feature genes in C2 are mainly related to tumor metastasis and immune regulation. These genes provide a solid foundation for constructing risk signatures. We further identified the prognostic feature genes of C2. Based on the research by Liu et al. (2022), we fitted these prognostic feature genes using a combination of 101 algorithms from 10 machine learning methods. Finally, we calculated the average C-index of the training set and validation set and determined that the RSF + Ridge algorithm combination can construct the best risk signatures. We defined these risk signatures as High-CAF Risk Signatures (HCAFRS). In both the training set and the three validation sets, the HCAFRS risk score was found to be an independent prognostic factor for CRC. It's worth noting that the Meta-Cohort, which integrates data from four groups, also showed the same trend. These findings indicate that HCAFRS has high reliability and stability. With the advancement of medicine, precision and efficiency are increasingly pursued. However, the commonly used TNM staging system for evaluating CRC prognosis and treatment is no longer sufficient to meet the needs of clinical physicians (Delattre et al. 2022). Therefore, in this study, we constructed a columnar chart model for predicting CRC survival based on HCAFRS and three commonly used clinical parameters (stage, age, and gender). Surprisingly, the calibration curve showed that the columnar chart model we constructed has very high stability, and DCA and time ROC curves indicated that the columnar chart model outperforms other clinical parameters. Therefore, HCAFRS is a promising alternative prognostic factor in clinical practice.

In subsequent studies, we found that the HCAFRS risk score was highly positively correlated with hypoxia, epithelial-mesenchymal transition, APICAL JUNCTION, angiogenesis, and myogenesis. Studies have shown that hypoxia promotes CRC metastasis, drug resistance, and immune escape, making it a driving factor in CRC progression (Zhou et al. 2022). Epithelial-mesenchymal transition and angiogenesis are also culprits in CRC progression (Shin et al. 2023; Hsu et al. 2023). CAFs also exhibit these biological features (Lavie et al. 2022), which may be because HCAFRS is constructed based on consensus clustering of differential genes in CAF subpopulation trajectories. Therefore, this study analyzed the relationship between HCAFRS and CAFs. Based on single-cell data, we found that the HCAFRS risk score mainly comes from CAFs, and combining bulk sequencing data showed that CAF infiltration is positively correlated with the HCAFRS risk score. Finally, spatial transcriptomics confirmed that the HCAFRS risk mainly originates from CAFs. Therefore, the HCAFRS constructed based on consensus clustering of differential genes in CAF subpopulation trajectories is reliable.

This study performed consensus clustering of CRC based on differential genes in CAF subpopulation trajectories (C1 and C2), revealing significant heterogeneity between C1 and C2, which was validated. We developed and validated High-CAF Risk Signatures (HCAFRS) based on the feature genes of C2. In conclusion, HCAFRS has the potential to serve as a promising prognostic indicator for CRC, improving the quality of life for CRC patients.

## Supplementary Information

Below is the link to the electronic supplementary material.

Supplementary file1 (DOCX 7305 KB)

Supplementary file2 (XLSX 72 KB)

## Data Availability

The datasets generated and analysed during the current study are available in the TCGA repository (https://portal.gdc.cancer.gov) and GEO database (https://www.ncbi.nlm.nih.gov/geo/).
