# Supplementary material for: Consensus clustering and development of a risk signature based on trajectory differential genes of cancer-associated fibroblast subpopulations in colorectal cancer
Source: J Cancer Res Clin Oncol. 2024 Aug 9;150(8):388. doi: 10.1007/s00432-024-05906-z (PMC11315798; doi:10.1007/s00432-024-05906-z)
Supplement: Supplementary file 1 — Supplementary file1 (DOCX 7305 KB) [file 432_2024_5906_MOESM1_ESM.docx]

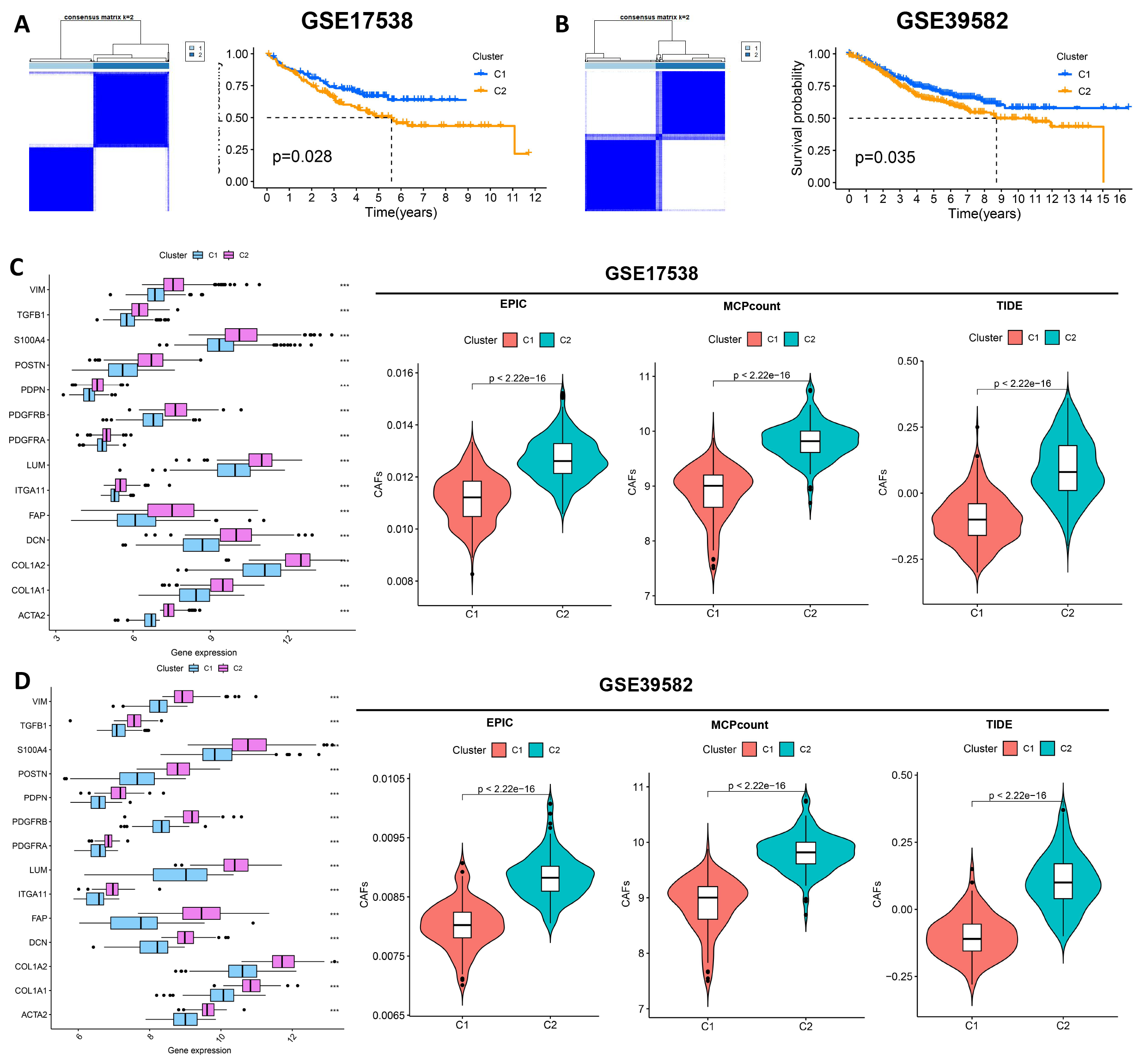


Supplementary Figure 1. Validation of CRC subtypes

A-B. CRC typing verification based on GSE17538 and GSE39528. B-C. Differential typing based on CAF labeling and CAF infiltration between GSE17538 and GSE3C1 and C2.


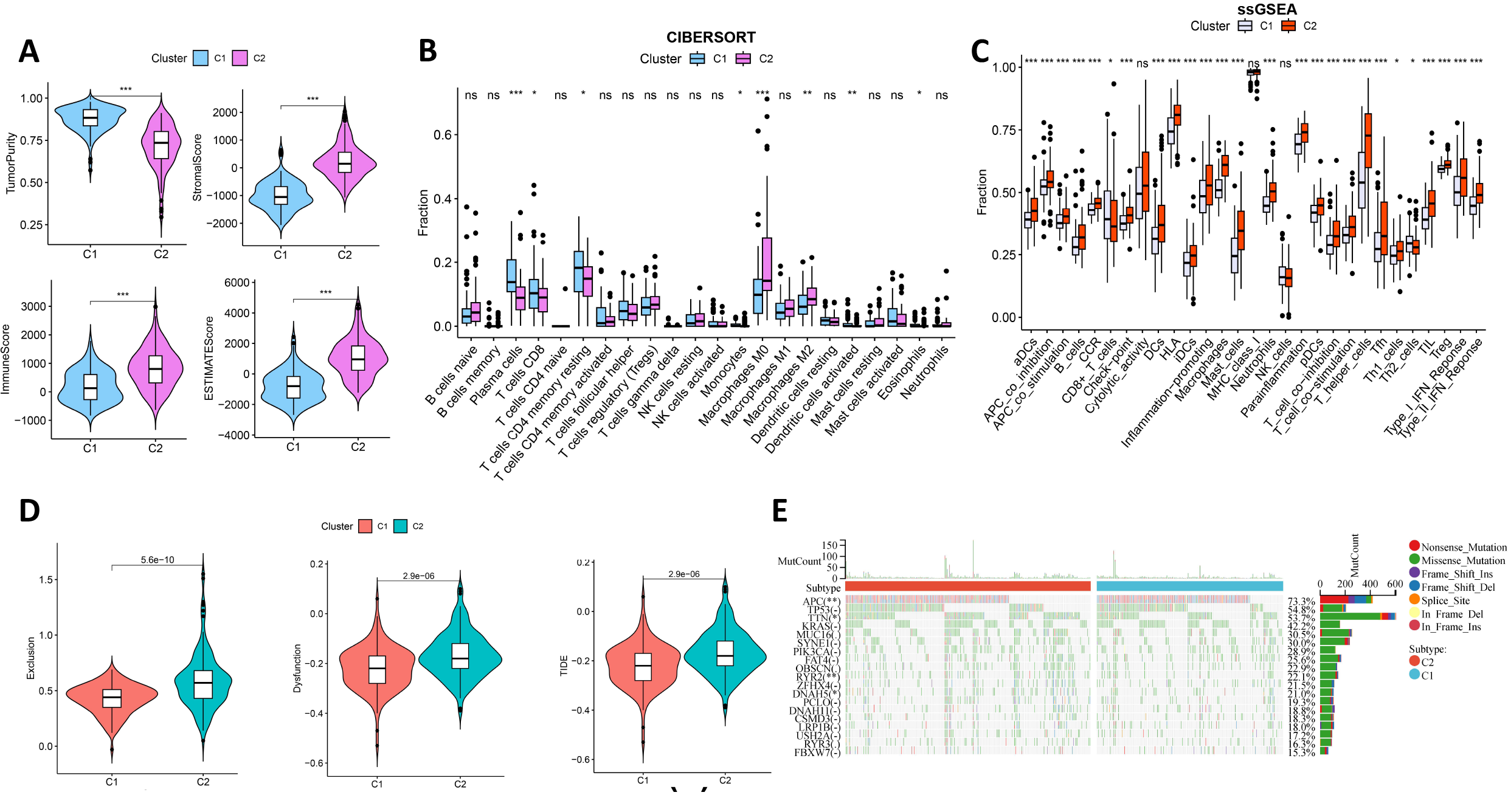


Supplementary Figure 2. TME, immune escape, and mutational differences between C1 and C2.

A. The violin plot shows the TME difference between C1 and C2. B. The box plot shows the difference in immune cell infiltration between C1 and C2. C. The box plot shows the infiltration scores of 16 immune cells and the activity of 13 immune-related pathways. D. The violin plot shows the difference in immune escape between C1 and C2. E. The waterfall plot shows the mutation analysis between C1 and C2.


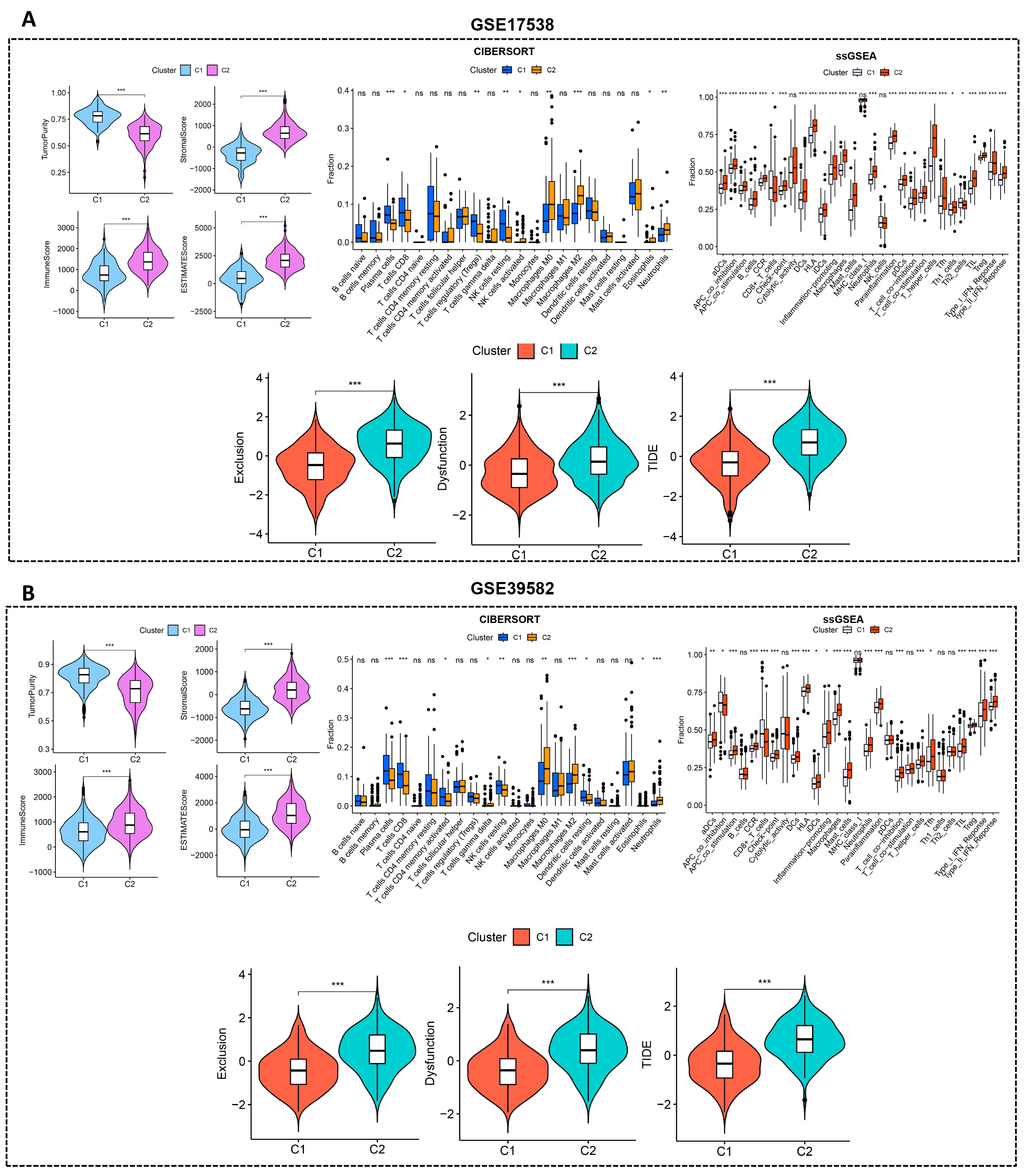


Supplementary Figure 3. Validation of TME and immune escape differences between CRC subtypes.

A-B. GSE17538 and GSE39582 CRC cohorts were used to validate differences in TME and immune escape between C1 and C2, respectively.


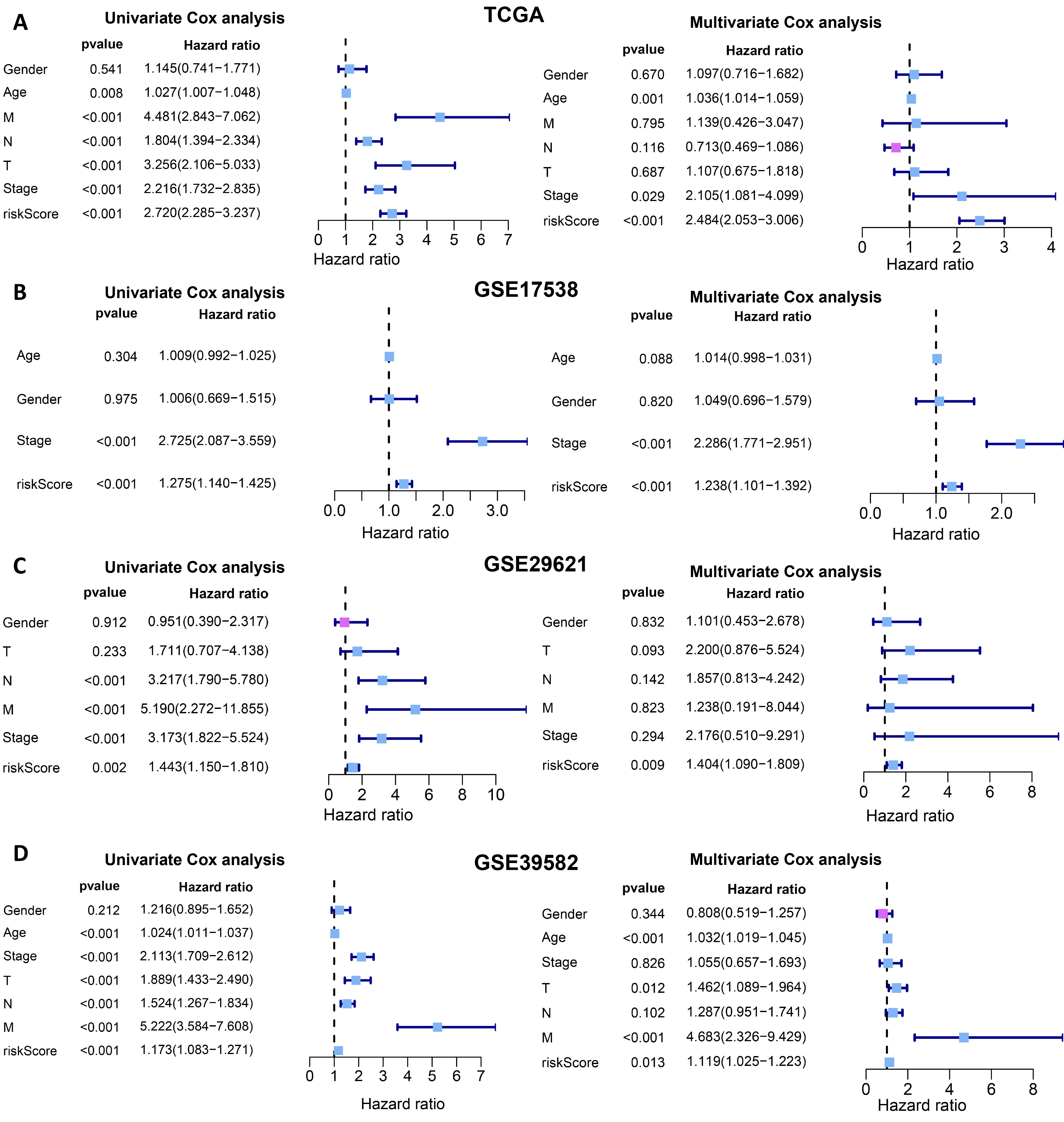


Supplementary Figure 4. Univariate/multivariate Cox analysis.

1. D. Univariate/multivariate Cox analysis of the training set (TCGA) and validation sets (GSE17538, GSE39582, GSE29621).


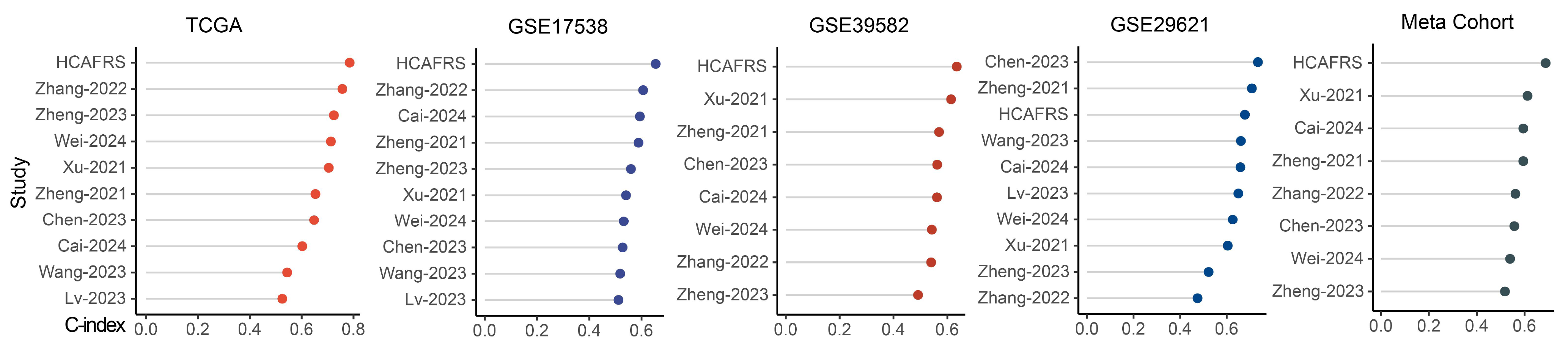


Supplementary Figure 5. Comparison of the C-index between this study (HCAFRS) and 9 published risk signatures.
